# COVID-19 Aftermath: Direction Towards Universal Health Coverage in Low-Income Countries: Comment on "Health Coverage and Financial Protection in Uganda: A Political Economy Perspective"

**DOI:** 10.34172/ijhpm.2022.7519

**Published:** 2022-09-14

**Authors:** Viroj Tangcharoensathien, Warisa Panichkriangkrai, Woranan Witthyapipopsakul, Walaiporn Patcharanarumol

**Affiliations:** International Health Policy Program, Ministry of Public Health, Nonthaburi, Thailand

**Keywords:** COVID-19, Universal Health Coverage, Low-Income Countries, Health Expenditure, Service Coverage, Unmet Healthcare Needs

## Abstract

Progressive realization of universal health coverage (UHC) requires health systems capacity to provide quality service and financial risk protection which supports access to services without financial hardship. Government health spending in low-income countries (LICs) has been low and heavily relied on external donor resources and out-of-pocket payment. This has resulted in high prevalence of catastrophic health spending or foregone care by those who cannot afford. Under fiscal constraints posed by pandemic, reforms in LICs should focus on efficiency through health resource waste reduction. Targeting the poor even with low level of health spending can make a significant health gain. Investment in primary healthcare and health workforce is the foundation for realizing UHC which cannot be postponed. Innovative tax on health hazardous products, conditional debt relief can increase fiscal space for health; while international collaboration to accelerate coronavirus disease 2019 (COVID-19) vaccine coverage can bring LICs out of acute phase of pandemic.

## Background

 The recent publication by Nannini et al on “Health Coverage and Financial Protection in Uganda: A Political Economy Perspective”^1^ highlighted a number of challenges in Uganda. From the article, the political context in Uganda was not conducive for universal health coverage (UHC). In 2010, the government policy shifted from social sector investment including health to growth-enhancing sectors such as infrastructure development. This resulted in deterioration of health services and shortage of essential medicines. The community-based initiatives were not supported by a clear policy direction.

 In Uganda, less than 2% of population were covered by financial risk protection systems. Financing had been relied on external donor sources and out-of-pocket payment; while government invested too little. The private health sector, accounting for half of the total providers was largely unregulated.

 In 2020, during the legislative process for a UHC Bill in Uganda, the Initiatives for Social and Economic Right submitted its comments and suggestions on the contents of draft Bill to the Parliament.^2^ Though the Uganda Parliament passed a National Health Insurance Bill on March 31, 2021, the President failed to ascent, but passed this back to the Ministry of Health for review and resubmission to Cabinet. Employers strongly opposed contribution to the National Health Insurance Scheme.^3^ We felt there is a long winding and rough road towards UHC in Uganda, especially complicated by the impacts of coronavirus disease 2019 (COVID-19) pandemic.

 Currently, there are 27 low-income countries (LICs), of which 23 are in Africa including Uganda. Similar to Uganda, all LICs have been struggling to make a good level of UHC service coverage and were further hit hard by the pandemic in the past few years. Instead of commenting on Uganda UHC, this paper provides broader perspectives and analyses the pandemic impacts and prospect to recovery and proposes strategic moves to ensure clear direction towards UHC in LIC settings in Africa.

## Health Development and Progress Towards Universal Health Coverage

 In Table, we compare key indicators in five selected LICs in Africa. Sustainable Development Goal (SDG) 3.8.1 indicator on UHC service coverage index ranged between 32 and 54 in these five countries. Within this composite index, we observed generally low level of modern contraceptive prevalence, very low skilled birth attendance in Ethiopia (28%) and Central African Republic (40%). The low coverage of skilled birth attendance resulted in high maternal mortality ratio in Central African Republic and Ethiopia. Nevertheless, DTP (diphtheria–tetanus–pertussis) immunization coverage was moderate and high except Central African Republic (42%).

**Table:** Key Statistics, Five Selected Low-Income Countries In Africa (Latest Available Years)

	**CAF**	**ETH**	**LBR**	**RWA**	**UGA**
I. Health status					
Mortality rate, under-5 (per 1000 live births) (2020)	103.0	48.7	78.3	40.5	43.3
Maternal mortality ratio (modelled estimate, per 100 000 live births) (2017)	829	401	661	248	375
II. Economic status					
GDP per capita (current US$) (2020)	481.8	936.3	601.1	786.3	822.0
GDP per capita growth (annual %) (2020)	-0.9	3.4	-5.3	-5.8	-0.4
Tax revenue (% of GDP) (2020)	8.7	6.7	N/A	15.1	11.4
Poverty headcount ratio at $1.90 a day (2011 PPP) (% of population)	65.9 (2008)	30.8 (2015)	44.4 (2016)	56.5 (2016)	41.0 (2019)
III. Health service coverage					
UHC service coverage index (2019)	32	38	42	54	50
Contraceptive prevalence, any modern method (% of married women ages 15-49)	14.4 (2019)	40.5 (2019)	23.9 (2020)	58.4 (2020)	36.5 (2018)
Births attended by skilled health staff (% of total)	40 (2010)	28 (2016)	84 (2020)	91 (2015)	74 (2016)
Immunization, DPT (% of children ages 12-23 months) (2020)	42	71	65	91	89
IV. Healthcare financing					
Current health expenditure (% of GDP) (2019)	7.8	3.2	8.5	6.4	3.8
Current health expenditure per capita (current US$) (2019)	37.2	26.7	52.6	51.4	32.4
Domestic general government health expenditure (% of current health expenditure) (2019)	10.6	22.7	16.1	40.0	15.1
Domestic private health expenditure (% of current health expenditure) (2019)	61.3	43.2	59.2	26.3	42.9
Domestic general government health expenditure (% of general government expenditure) (2019)	4.8	4.8	4.1	8.9	3.2
External health expenditure (% of current health expenditure) (2019)	28.1	34.1	24.7	33.8	42.0
Out-of-pocket expenditure (% of current health expenditure) (2019)	60.3	37.9	54.4	11.7	38.3
Proportion of population spending more than 10% of household consumption or income on out-of-pocket healthcare expenditure (%)	6.8 (2008)	2.1 (2015)	6.8 (2016)	1.2 (2016)	15.3 (2016)
V. Health workforce					
Physicians (per 1000 people)	0.1 (2015)	0.1 (2018)	0.04 (2015)	0.1 (2019)	0.2 (2017)
Nurses and midwives (per 1000 people)	0.2 (2015)	0.7 (2018)	0.5 (2018)	1.0 (2019)	1.2 (2018)
Doctors, nurses and midwives (per 1000 people)	0.3	0.8	0.6	1.1	1.4

Abbreviations: CAF, Central African Republic; ETH, Ethiopia; LBR, Liberia; RWA, Rwanda; UGA, Uganda; UHC, universal health coverage; DPT, diphtheria–tetanus–pertussis; GDP, gross domestic product; PPP, purchasing power parities. Source of data: World Development Indicators, the World Bank (https://data.worldbank.org/indicator).

 Per capita current health expenditure was lower than US$ 53 in all five countries; indicating inadequacy to respond to health need of population. In Uganda, current health expenditure relied on external donor (42%) and private sources (43%) notably out-of-pocket payment by households (38%). Government health expenditure was below 5% of government expenditure in all countries except Rwanda (8.9%). Prevalence of catastrophic health expenditure was high in all countries except Rwanda (1.2%) and Ethiopia (2.1%). The low UHC service coverage index can be explained by services unavailability, or services not being used due to unaffordability, unawareness, lack of trust on the demand side; geographical inaccessibility, limited capacity on the supply side. The low prevalence of catastrophic health expenditure represents good outcome when people have adequate access to care. However, low catastrophic prevalence attributed from foregone care must be rectified. This calls for regular monitoring of unmet healthcare needs.

 Using UHC service coverage index below 50 and a density of doctors, nurses and midwives below the global median of 48.6 per 10 000 population, the World Health Organization (WHO) has classified 47 countries worldwide as critical shortage that should be safeguarded from international recruitment.^4^ All these five countries except Rwanda belong to this group, having the density between 0.3 and 1.4 per 10 000 population. There remain considerable gaps to meet the threshold set by WHO African Region which recommends 134 per 10 000 population of 13 cadres of health workforce to attaining at least UHC service coverage index of 70.^5^ The current health workforce density was far below the threshold for achieving health-related SDG of 44.5 doctors, nurses, and midwives per 10 000 population.^6^

## COVID-19 Pandemic Impacts and Prospect to Recovery

 LICs have very limited fiscal space to confront the pandemic, finance COVID-19 vaccines and support health systems recovery. As of June 30, 2022, 18.5% of population in Africa had at least one dose of COVID-19 vaccine, while this was 75.6% in high-income countries.^7^ Vaccine roll-out in Africa is hampered by failure of global vaccine solidarity, affordability and vaccine hesitancy,^8^ and capacity to immunize large population.^9^ As affected by pandemic, the data from 2020 revealed that the annual per capita gross domestic product (GDP) growth were all negative except Ethiopia (3.4%); and low fiscal space as government tax revenue was 7-8% of GDP in Central African Republic and Ethiopia. People living below the absolute poverty ($1.90 per capita pe day) was high, 65.9% and 56.5% in Central African Republic and Rwanda (see Table). Resources for pandemic response were shifted from other critical areas including education and public investment. Unsustainable external debt burdens, additional borrowing during the pandemic and increasing debt-servicing cost pushed a number of LICs on the verge of debt crisis. This requires urgent coordinated international support for debt relief.

 Evidence shows pandemic recovery with higher levels of inequality within and between countries—a scar of the pandemic. The per capita GDP in developed economies is expected to fully recover by 2023; while it is elusive for many developing countries. Developing countries in Africa foresee a gap of 5.5 percentage points of GDP per capita compared to pre-pandemic projections.^10^

 LICs especially in Africa also foresee a slow recovery of jobs; the pace of job creation does not match with the earlier employment losses and new entry into labour force. This will result in increased number of people living in extreme poverty above the pre-pandemic levels. The absolute number of people living in poverty in African LICs will continue to rise through 2023.

 Complex humanitarian emergencies and natural disasters pose significant challenges to vulnerable populations in Africa. Millions of people in West and Central Africa were driven to the edge of survival due to a confluence of factors, such as conflict and violence, extreme poverty, weak governance, chronically high food insecurity and malnutrition, and the impact of climate change.^11^ The March-May 2022 rainy season was the driest on record in Africa, causing acute food insecurity and rising malnutrition in Ethiopia, Kenya and Somalia.^12^

## Strategic Direction Towards Universal Health Coverage

 The gloomy economic recovery, rampant poverty, inequality, complex humanitarian emergencies, low vaccine coverage, fragile health delivery systems can offset the progress of health development and offtrack many health goals in the SDG. Strategic moves to set the direction towards UHC require political leadership across successive governments which are guided by solid evidence.

 A study by WHO indicates that when health spending increases, UHC coverage index also increases. Notably, increased per capita public health spending (between purchasing power parities $40 and 60) improves service coverage significantly, but not financial risk protection which only improves significantly when public spending is greater than purchasing power parities $200 per capita. Adequate absolute level of public spending on health is critical for UHC progress. However, at any given level of spending, particularly at low levels, there are certain scopes to focus on greater efficiency.^13^

 WHO estimated 20% to 40% of total health spending was wasted through inefficiency.^14^ It proposed ten areas for efficiency gains especially in hospital sector which consumed most healthcare resources. Key policy interventions should focus on medicines such as the increased use of quality generic medicines, controlling excessive mark-up, surveillance and legal sanctions against substandard and falsified medicines. The unethical market promotion by pharmaceutical industry triggers inappropriate use, wasteful over-prescribing and dispensing.^15^ Fee-for-service payment stimulates supplier-induced demand^16^ or fear for litigation leads towards defensive medicines which drive healthcare cost without clinical benefits.

 A study by Sparkes et al^17^ proposed two-pronged strategies. The first is collaboration beyond health sector such as working with Finance Ministry to introduce innovative tax on health-harmful products such as tobacco, alcohol and sugary beverage; conditional debt relief package upon governments increased spending on health and education and timely humanitarian assistance where needed.

 Second, health policy needs to prioritize the poor and vulnerable. General tax financed UHC has more comparative advantage than payroll tax in which entitlement terminates when members are jobless from pandemic. Sparkes and colleagues also advocated monitoring of foregone care which might result in low prevalence of catastrophic health spending. Efficiency gains through provider payment reform towards closed-ended such as capitation and Diagnostic Related Group, even at the low level of health spending can make a difference on UHC coverage index and financial risk protection. Provider payment reform synergizes with the use of generic essential medicine and regulatory measures against supply-induced demand can improve efficiency significantly. Long term investment to strengthen and extend primary healthcare covering the whole population is the foundation for successful UHC implementation.^18^

## Conclusion

 Even in fiscal constraints from pandemic and current low health spending, governments in LICs can seize the opportunity to minimize resource waste, increase efficiency and prioritize the poor and most vulnerable while in parallel lay down a firm foundation of primary healthcare infrastructure and health workforce as a platform for implementing UHC. We urge Inter-agency and Expert Group on SDG indicators to consider a new indicator of 3.8.3 on prevalence of unmet healthcare needs which is a flip side of SDG 3.8.2 on prevalence of catastrophic health spending.

## Acknowledgements

 We thank Waritta Wangbanjongkun for her contribution in retrieving essential data in Table.

## Ethical issues

 Not applicable.

## Competing interests

 Authors declare that they have no competing interests.

## Authors’ contributions

 Conceptualization WPan, WW, WPat, and VT; writing original draft preparation VT; writing review and editing WPan, WW, and WPat, VT. All authors have read and agreed to the published version of the manuscript.
